# The Development of Subitizing in Bilingual Children

**DOI:** 10.1111/desc.70296

**Published:** 2026-11-03

**Authors:** Elisabeth Marchand, Nina Schoener, Jocelyn Sandoval‐Franquez, Kelly Kendro, David Barner

**Affiliations:** ^1^ University of California San Diego USA; ^2^ Fisher College Boston Massachusetts USA; ^3^ University of California Berkeley California USA; ^4^ Northern Arizona University Flagstaff Arizona USA

**Keywords:** bilingualism, cardinality, cognitive development, counting, number words acquisition, subitizing

## Abstract

What role does language‐specific experience play in the development of numerical knowledge? Previous studies have probed this question by testing bilingual learners, who have different linguistic representations of number across their two languages. Here, we take this approach to study the effect of language‐specific experience on children's ability to rapidly estimate small sets (i.e., subitizing). We tested 66 Spanish–English and German–English bilinguals, aged 3–6, and found that bilinguals made more accurate verbal estimates of small sets in their dominant number language (i.e., the language in which they could count the highest). This was despite the fact that all children in the study were able to accurately count much larger sets, and were therefore classified as “cardinal principle knowers” in both of their two languages. These results provide evidence for early emerging individual differences in estimation abilities that are due to linguistic experience. Also, they suggest that even after children have begun to learn more advanced skills (e.g., accurate counting of large sets), individual differences in earlier learned skills (e.g., subitizing of small sets) may persist. Different language‐specific experiences may therefore impose persistent effects on numerical development, with implications not only for bilingual learners, but also for monolinguals with impoverished exposure to number language.

**Summary:**

- Subitizing skills differed across bilingual children's two languages, with stronger mappings in the language in which they can count the highest.
- Bilingual children's subitizing skills in their dominant language, but not their nondominant language, were similar to the subitizing skills of monolinguals.
- Cross‐linguistic differences found for small numerosities did not extend to larger sets.
- Disparity in knowledge of number word meanings found in bilingual subset‐knowers persisted even after children could accurately count large sets and become CP‐knowers.

## 1 Introduction

Although many animals can represent approximate numerical magnitudes, only humans have symbols for large exact numbers, a fact that is often attributed to our species‐specific capacity for natural language (Carey and Barner 2019; Gordon 2004; Le Corre and Carey 2007; Pica et al. 2004; Spaepen et al. 2013; Spelke 2017). However, the precise role that language plays in the acquisition of exact number remains unclear, in part because linguistic and non‐linguistic capacities emerge and change together during development, making their respective roles difficult to disentangle. In efforts to isolate the role of language from non‐linguistic factors, recent studies have focused increasingly on cross‐linguistic comparison, as well as on studies of bilingual learners, who make it possible to distinguish between language‐specific knowledge and knowledge that is not specific to a particular language. In the present study, we adopted this logic and investigated language‐specific processes involved in subitization, a mechanism that has been argued to play a fundamental role in building exact representations of numbers.

Previous studies of bilinguals have played an important role in revealing how some forms of numerical abilities rely on language‐specific experiences. For example, in one study, Spelke and Tviskin (2001) trained English–Russian bilinguals in each of their two languages on exact and approximate calculations (e.g., additions in base 6, cube root estimation). They found that when bilinguals were trained on problems in one language, they were slower and less accurate when solving similar problems in their second language, but only when the calculations involved exact numbers. In contrast, approximate calculations were solved equally well in their two languages. Similar findings have been observed with other forms of exact arithmetic (e.g., multiplication and subtraction) and in bilingual speakers of different languages (Saalbach et al. 2013; Van Rinsveld et al. 2015; Venkatraman et al. 2006). Behavioral findings have also been corroborated by neuroimaging techniques, which show that exact computations, unlike approximate calculations, systematically recruit language areas (Grabner et al. 2012; Lin et al. 2012; Mondt et al. 2011; Salillas and Wicha 2012). Overall, these findings suggest that numerical skills that draw on stored math facts and exact computations are not only language‐dependent but also language‐specific, and don't readily transfer across languages.

While previous studies have found evidence for the role of language‐specific experiences in arithmetic, less is known about how bilinguals represent other forms of numerical knowledge, and how these representations emerge in development. For example, considerably less is known about earlier learning processes in bilinguals, including how initial exact meanings are learned, and how these early linguistic expressions of exact number become associated with approximate number representations. In particular, little is known about whether these forms of knowledge transfer automatically across languages, or if learning in a child's two languages is independent. In one recent study of this question, Marchand et al. (2020) investigated the estimation abilities of 5‐ to 7‐year‐old bilingual children. They found that bilinguals estimated arrays differently across their two languages—making more accurate estimates in their more dominant language than in their less dominant one (as determined by how high they could count in each language). Although many of these children could count higher in one language than in the other, the reported difference in estimation accuracy was found even for numbers that children could produce in both languages, indicating that estimation differences weren't simply due to lacking words in one of the two languages.

These findings regarding estimation in bilinguals support two conclusions. First, they show that changes in estimation skills in early childhood do not depend purely on the development of non‐verbal number representations. Second, these results show that linguistically‐mediated disparities in numerical knowledge—which presumably can explain individual differences between monolinguals too—begin to emerge relatively early in development, by at least 5 years of age. Critically, however, the results of Marchand et al. (2020) leave open when this linguistically‐mediated disparity in estimation ability first begins to emerge. This is important because some theories have argued that children begin number word learning by associating individual words like “one”, “two”, and “three” with cardinalities (Carey 2004). However, Marchand et al. tested larger numbers (i.e., 8 and higher), and the youngest children were 5 years of age. Therefore, in the current study we investigated the earliest moments of number word acquisition in bilingual children to assess when disparities in estimation abilities might first emerge in development, focusing on numbers within the subitizing range of 1–3. In this way, we asked when language‐specific experiences begin to impose different learning trajectories on number knowledge.

When monolingual children learn number words, they typically go through a phase during which they can recite part of the count list (e.g., number words *one* through *five*) without understanding the meanings of individual words, similar to rote knowledge of songs or the alphabet. However, not long after, they begin to build associative mappings between small number words and cardinalities. Using Wynn's (1990, 1992) Give‐a‐Number task (Give‐N), studies in this literature have found that children begin by associating the word *one* with sets containing exactly one object: when asked to give *one* object, they correctly provide one object, but can't reliably give correct amounts when asked to give larger numbers. For this reason, children at this stage are often called “one‐knowers”. Some months later, children learn the meaning of *two*, and respond correctly when asked to give *one* or *two* objects, but not larger numbers. These children are called “two‐knowers.” Following the same pattern, children become “three‐knowers” and sometimes “four‐knowers” over a period of many months. Finally, after moving through these “subset” stages, children seem to realize that they can use the counting procedure to generate sets of different sizes, and use the counting routine that they learned much earlier to accurately give sets larger than 3–4. Since children at this stage seem to understand that counting relates to cardinalities, they are called Cardinal Principle or Counting Principle knowers (CP‐knowers; for discussion of these stages and their interpretation, see Cheung et al. 2017; Davidson et al. 2012; Schneider et al. 2020; Sella and Lucangeli 2020; Sella et al., 2020). This general pattern of development has been found in many studies across many different languages (Almoammer et al. 2013; Barner et al. 2009; Ceylan and Aslan 2018; Condry and Spelke 2008; Jara‐Ettinger et al. 2017; Le Corre et al. 2016; Li et al. 2003; Marchand and Barner 2019; Meyer et al. 2020; Negen and Sarnecka 2012; Nikoloska 2009; Piantadosi et al. 2014; Sarnecka et al. 2007; Sarnecka et al. 2018; Schneider et al. 2020; Wagner et al. 2015; Wynn 1990, 1992; see Wege et al. 2025 for a review).

While this process of associating number words with cardinalities has been well documented in monolingual children, less is known about this early learning process in bilingual learners. In one study of this question, Wagner et al. (2015) tested French–English and Spanish–English bilingual children in their two languages on the Give‐N task and found that bilinguals had different knower levels in their first (L1) and second (L2) languages. In particular, they found that although children classified as CP‐knowers in one language were generally CP‐knowers in their other language too, a different pattern was found for children who could not yet accurately count: children who were identified as 1‐, 2‐, or 3‐knowers in one language had a different knower level in their second language about 2/3 of the time. For example, most children who were classified as, for instance, 2‐knowers in the L1 were not classified as 2‐knowers in their L2, despite being able to count well past *two* in both of their two languages. Similarly, results have also been reported in subsequent studies, spanning multiple languages. First, a study by Sarnecka et al. (2023) compared the early number knowledge of Spanish–English bilinguals across their two languages and found that children performed significantly better in English (their language of instruction) across multiple tasks (i.e., counting to 10, counting 6 objects, and the Give‐N task). Second, Alhaider et al. (2024) found that English–Arabic bilingual children learned the word for 2 earlier in Arabic than in English, perhaps due to the presence of dual grammatical morphology in Arabic (see also Almoammer et al. 2013; Marušič et al. 2016, 2021). Finally, Cheung et al. (2025) used the Give‐N task to test Singaporean children aged 2 to 6 who were bilingual in English and Mandarin, Malay, or Tamil and found subset‐knower had weak but significant correlations between knower levels across their languages, while cardinal principle knowledge was highly related across languages, as in Wagner et al. (2015).

Together, these studies paint a nuanced portrait, in which knowledge of cardinal principle knowledge appears to transfer across languages in bilingual learners, whereas knowledge of smaller number word meanings may differ before children become CP‐knowers. Interestingly, however, these studies leave open how children represent small number words across their languages after they have learned the cardinal principle. Although bilingual children exhibit different knowledge of small number words prior to learning to count, this difference between languages appears to resolve itself when children learn to count and become CP‐knowers: when children become CP‐knowers in one language, they appear to automatically become CP‐knowers in their second language. This is interesting because it raises the possibility that when bilingual children become CP‐knowers, their knowledge of small number words across their two languages might equalize, since they can now give small and large numbers in both languages upon request.

Compatible with this logic, to be classified as a CP‐knower in the Give‐N task, children must typically provide accurate responses not only to larger numbers, but also to small ones. We hypothesized that any such differences in mapping strength arise from differences in the amount of number‐specific input bilingual children receive in each of their two languages, such that stronger word‐cardinality associations develop in a child's more dominant number language (see General Discussion for further consideration of this mechanism and of alternative accounts). However, an alternative hypothesis is that, while bilingual children may have reached the same minimum standard to be classified as CP‐knowers in both languages, they may still exhibit language‐specific differences in how robustly they represent small number words, in ways not detectable by the Give‐N task. Critically, to be credited with knowledge of a number word like “two” on the Give‐N task, a child needs only to respond correctly on 2/3 of requests to give this number. Consequently, a child who has very strong associative mappings between small number words and cardinalities in one language but relatively weak associations in their second language might still be credited with the same knowledge of these words in their two languages, so long as their associations are strong enough to respond correctly on 2/3 trials in each case. Further, given the finding that counting abilities transfer across a child's two languages, it's also possible that bilinguals respond successfully to requests for smaller numbers in their weaker language without drawing on associative mappings at all, and simply count when asked to give smaller sets, thereby masking the differences in knowledge across their two languages.

One way to address this issue is to assess bilingual children's knowledge of small number words in a task that does not permit counting, via tests of “subitizing” (Kaufman et al. 1949; Mandler and Shebo 1982; Starkey and Cooper, 1995). Typically, in a subitizing task, participants are presented with flashed arrays of dots (1 to 4 dots) and are asked to make verbal numerical estimates of the cardinalities. Due to the rapid presentation of stimuli, the task discourages counting, such that participants must rely instead on associations between words and sets. A subitizing task might also be more sensitive to small differences in knowledge than Give‐N because it can be used to measure not only accuracy on individual trials, but also differences in response time. Finally, beyond the benefit of having a stronger measure of the mappings between number words and cardinality, studying knowledge of subitizing skills across languages in bilinguals is on its own important because subitizing performance has been shown to be correlated with non‐symbolic arithmetic, number line estimation, and counting (LeFevre et al. 2010, LeFevre et al. 2022) and has been argued to play an important role in the acquisition of number words (Carey 2004).

In the current study, we tested bilingual CP‐knowers’ subitizing abilities across their two languages to investigate the role of language‐specific experiences in the acquisition of number words. To assess generalizability across different language and SES groups, we tested two samples of bilingual children: Spanish–English children from low SES families, and German–English bilinguals from high SES families. If bilinguals, in the process of becoming CP‐knowers, build equally robust mappings between number words and their cardinality across languages, then we should not find differences in subitizing abilities across languages. However, if these mappings still remain reliant upon language‐specific experiences across languages even after children become CP‐knowers, then we should see differences in subitizing performances across languages.

## 2 Method

All data and model outputs are available here: https://osf.io/gwz28/overview?view_only=0a7ad5625a544c3bbc30d1647cf862bb. All planned analyses were preregistered and were conducted as planned unless otherwise stated.

### 2.1 Participants

We tested a total of 113 bilingual children. Forty‐seven children were excluded from analysis because of (1) being a subset‐knower (*n* = 20), (2) reaching the same highest count in each language (*n* = 5), (3) language delay (*n* = 3), (4) failure to complete all 6 tasks (*n* = 8), and (5) experimenter error, parental interference, or technological issues (*n* = 11). Our final sample included 66 children (36 Spanish–English and 30 German–English bilinguals), aged 3.47–5.98 years (*M* = 4.98 years). Additionally, we tested 73 English monolingual children for comparison. Thirty‐one children were excluded from analyses because of (1) being a non‐knower or subset‐knower (*n* = 17), (2) failure to complete all 3 tasks (*n* = 6), or (3) experimenter error, parental interference, or technological issues (*n* = 8). Our final sample of monolingual children included 42 participants, aged 2.75–5.87 (*M* = 4.50 years). All monolingual and bilingual participants were recruited from either a participant database, via communications with preschools, or using online recruitment tools such as Facebook and Children Helping Science. All participants lived in the United States, with most living in California. The majority of German–English bilingual participants were recruited from private German‐speaking daycare centers or schools; though we did not collect detailed socioeconomic information, most of these participants were living in affluent areas in California (e.g., Silicon Valley). The majority of Spanish–English bilingual participants were recruited both through word‐of‐mouth and through primarily Hispanic public school districts in California; these districts had average median household incomes below the average for California according to the 2021 Census Reporter (U.S. Census Bureau 2021). Recruitment was performed by either German–English or Spanish–English bilingual research assistants. As part of the sign‐up and consent process, parents received a short questionnaire with questions about the child's first language, second language, and whether the child was bilingual. Children who were not identified as bilingual by caregivers were excluded from the bilingual sample and took part in the study as monolinguals. For all analyses, we determined the language dominance of participants based on their numerical knowledge rather than their non‐numerical linguistic knowledge. This decision was motivated by previous work showing that a child's L1 is not necessarily their dominant number language (e.g., Wagner et al. 2015), especially in cases where primary formal education is in their L2.

### 2.2 Materials and Procedure

Since this study took place during the COVID‐19 pandemic, all participants were tested online via Zoom. Bilingual children received two blocks of tasks—an English block and a Spanish or German block—the order of which pseudorandomly varied between participants to counterbalance language presentation. Within each block, children were always presented with 3 tasks in the same order: (1) Give‐a‐Number task, (2) Fast Cards, and (3) Highest Count task. Both groups of bilinguals (Spanish–English and German–English) were tested by an L1 speaker of either German or Spanish who also spoke English. The testing session lasted approximately 30 min. The procedure for monolingual children was identical to that of bilinguals except that they were administered only the English block of tasks and their testing session lasted approximately 15 min. Monolingual children were tested by an L1 English speaker. All children received a small prize for their participation at the end of the testing session.

### 2.3 Give‐A‐Number Task

This task was adapted from Wynn (1990), and its goal was to determine whether children were CP‐knowers or not, since only CP‐knowers were included in our main analyses. Before the testing session, parents were asked to gather a container (usually a plate) and 10 small identical objects (e.g., almonds, coins, raisins, etc.). For bilinguals, the task was administered in both of the child's two languages, and the experimenter began the task by addressing the child in the language to be assessed first (e.g., English, German, or Spanish). The experimenter first asked the child to put a certain number of objects into the container (e.g., “Can you put three almonds on the plate?”). After this first prompt, children were asked to count to verify that they had provided the right number, and if they chose to fix their answers, only final responses were recorded (Gibson et al. 2019). The trial structure followed a titration procedure (Marchand et al. 2022). Children were always asked for one first, and then two, and if they succeeded on both trials, the experimenter then asked for three. Otherwise, they asked for one. The subsequent requests depended on the child's pattern of response: if the child succeeded on a trial requesting N, the experimenter asked for N+1, but if the child failed, they asked for N‐1. The highest request was six. Participants were credited as CP‐knowers if they correctly gave N objects at least 67% of the time when asked for N (and not other requests) for numbers up to 6. Otherwise, they were classified as subset knowers. Bilingual children were tested by the same experimenter in both languages. Only children classified as CP‐knowers in both languages were included in the main analyses.

### 2.4 Fast Cards Task

This task was adapted from Le Corre and Carey (2007) and was used to assess children's subitizing abilities. Participants were presented with dot arrays ranging in magnitude from 1 to 8. Critical trials tested sets of 1–4, and additional trials tested sets of 6 and 8. The larger sets were included in exploratory analyses to assess children's mapping to large numbers. For each language that a child spoke (e.g., English and Spanish), all magnitudes were presented 4 times each, for a total of 24 trials per language. The trials were divided into four blocks containing one presentation of each of the six magnitudes. Within each block, the order of trials was pseudo‐randomized such that it differed across blocks. Two fixed block orders were created. For all participants, half of the trials controlled for total surface area while the other half controlled for dot diameter. In the trials controlling for total surface area, the total surface area occupied by the dots was held constant, whereas in the trials controlling for diameter, the diameter of the dots was held constant. Two fixed orders of the controlled parameters were created: for half of the participants, the first trial controlled for diameter (and alternated thereafter with total surface area), whereas for the other half of participants, the first trial controlled for total surface area. Bilingual participants completed the task in both languages, making language (English vs. Spanish/German) a within‐subject factor. To counterbalance potential order effects, half of the bilingual children completed the English block first, and the other half completed the Spanish/German block first. Language order was determined by the counterbalancing scheme and was independent of children's language dominance. Dots were displayed on the screen for 1 s. All dots were white and displayed on a black background. The instructions were provided to children in the language dictated by the testing condition (Spanish/German or English depending on the language being tested), or in English for monolingual participants. Participants were instructed to look at the dots and guess how many there were: “*Let's play a guessing game! Some dots are going to flash on the screen for just a second. I want you to guess how many dots there are! Ready? Great! How many dots did you see on the screen?*” Two practice trials were modeled by the experimenter (with sets of 5 and 7) to ensure that children understood that they needed to guess without counting. Noninformative verbal encouragement was given to children to keep them motivated, regardless of response accuracy (e.g., “*That's a good guess!*”).

### 2.5 Highest Count Task

This task had two goals. First, following Wagner et al. (2015), we used it to identify bilingual children's dominant number language. Second, we used children's highest count as a general proxy for counting experience. Children were asked to count a set of 30 cartoon cats presented on a PowerPoint slide, arranged in four rows of approximately eight cats. For both monolinguals and bilinguals, counting was recorded up to a shared ceiling of 30, well above the count level required for CP‐knower classification. Since bilingual children's Highest Count in each language was also used to classify their dominant number language, bilingual children who reached this ceiling were prompted to continue counting further so that ties between languages—which would have left this classification ambiguous—could be resolved. Bilingual children's dominant number language was defined as the language in which they were able to count highest before stopping or making an error. Below, we refer to the dominant number language as their NL1 and the nondominant number language as their NL2. For monolingual children, for whom no cross‐language classification was needed, no further prompting occurred once the ceiling was reached; this ceiling was well above the count level required for CP‐knower classification and therefore did not affect knower‐level determination.

## 3 Results

For all analyses, we used mixed effects logistic regression models constructed in R using the *lme4* package version 1.1‐27.1 (Bates et al. 2015).

### 3.1 Highest Count

We first compared bilingual children's highest counts across their two languages to identify their dominant Number Language or NL1 (see Figure 1). Their nondominant language was labeled their NL2. Among the 36 Spanish–English bilingual children, 20 counted higher in English and therefore their NL1 was identified as English. Among the 30 German–English bilinguals, English was the NL1 for 18 children. Notably, in both groups, a majority of children were English‐dominant for counting (Spanish–English: 20/36, 56%; German–English: 18/30, 60%), despite attending Spanish‐ or German‐language instruction (consistent with Wagner et al. (2015)) and suggesting that bilingual children in the US may receive substantial counting exposure outside formal schooling. On average, both Spanish–English and German–English bilinguals counted slightly higher in English than in their other language (for Spanish–English: *M*_English_ *=* 20.8, *range =* 5–100; *M*_Spanish_ *=* 15.8, *range =* 5–59; for German–English: *M*_English_ *=* 22.3, *range =* 5–35; *M*_German_ *=* 18.8, *range =* 11–32). Children had a mean highest count of 24.2 in their NL1 (*range =* 6–100) and 14.4 in their NL2 (*range =* 5–59), which was significantly different (*V* = 2211, *p* < 0.001).

**FIGURE 1:**
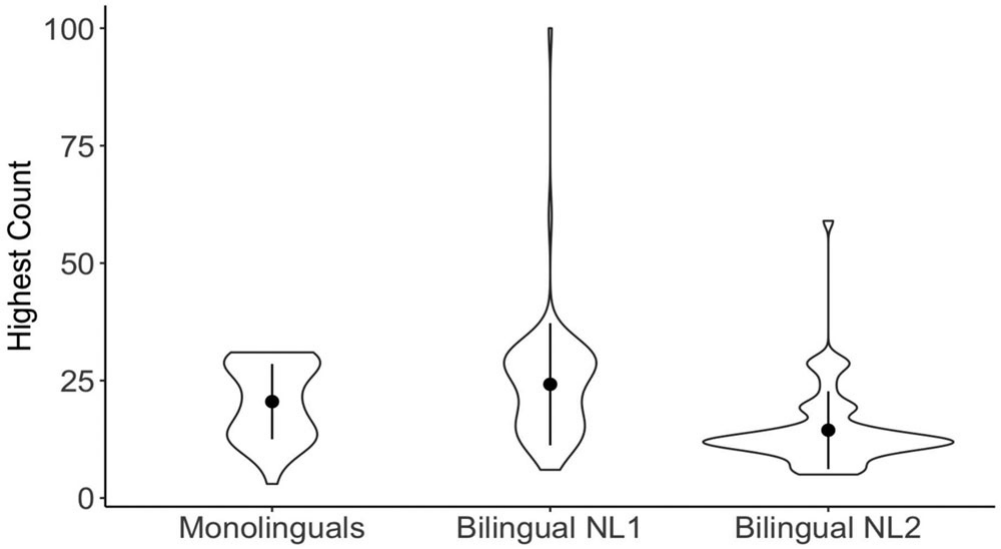
Participants’ Highest Counts by dominant language. *Note*: NL1 stands for children's dominant language, and NL2 is their less dominant language. There were 38 English‐dominant, 12 German‐dominant, and 16 Spanish‐dominant children.

Children in our monolingual sample had highest counts similar to the NL1 counts of our bilingual children (*range =* 3–31; *M =* 20.5), and the difference between these two datasets was not statistically significant (*p* = 0.07). However, the highest count of monolinguals did differ significantly from the NL2 highest counts of bilinguals (*t*(90) = −3.80, *p* < 0.001).

### 3.2 Subitizing of Bilinguals Across Languages

Before conducting our main analyses comparing the subitizing abilities of bilingual children across their two languages, we excluded all responses that were not provided in the format of a unique verbal estimate (*n =* 50/3168), for example, “too many” or “poquitos”. Responses that were 10 times larger than the mean estimate for each numerosity presented were also excluded, as pre‐registered (*n =* 17/3168). All analyses were performed on the remaining 3101 responses.

Next, we conducted preliminary analyses to test whether there were any effects of Task Order or Bilingual Type (i.e., German–English vs. Spanish–English bilinguals). To do this, in separate analyses, we predicted Accuracy of responses (coded as 0 or 1) from Task Order and Bilingual Type, and in both cases, Participant was coded as a random factor. Since we found no effect of Task Order or Bilingual Type (all *p*s > 0.10), these factors were excluded from subsequent analyses and all bilingual children were analyzed together.

### 3.3 Main Analyses

In our main analyses, we were interested in whether bilingual CP‐knowers subitized differently across their two languages (see Figure 2). We analyzed responses for numerosities 1 to 4 using model comparison. In our first model, we predicted Accuracy (coded as 0/1) from Age (in years) and Numerosity (1–4), with Participant coded as a random factor. In this model, both Age (*Estimate* = 1.32; SE = 0.30, *Z* = 4.43, *p* < 0.001) and Numerosity (*Estimate* = −1.62; SE = 0.12, *Z* = −13.29, *p* < 0.001) were significant. As expected, Accuracy improved as a function of Age and decreased as the Numerosity of sets increased. In our second model, we added the effect of Number Language (i.e., NL1 vs. NL2) to a model that otherwise was identical to the first model. In this second model, Age (*Estimate* = 1.34; SE = 0.30, *Z* = 4.43, *p* < 0.001) and Numerosity (*Estimate* = −1.63; SE = 0.12, *Z* = −13.30, *p* < 0.001) remained significant. Importantly, we also found that Number Language was significant (*Estimate* = −0.57; SE = 0.18, *Z* = −3.16; *p* = 0.002). An ANOVA comparing these models found that adding Number Language significantly contributed to the explanatory power of the model (*p* < 0.01), suggesting that bilingual CP‐knowers subitized differently across their two languages. In particular, as can be seen in Figure 2, bilingual children subitized more accurately in their NL1 relative to their NL2. As this analysis treated dominance as a categorical variable, in a follow‐up analysis we computed two continuous measures of dominance for each child (i.e., the difference and the ratio between their NL1 and NL2 Highest Counts) and added each to the model in turn. These continuous measures confirmed that the effect of Number Language did not depend on our binary classification of dominant versus nondominant language: neither measure improved model fit as a main effect, nor did it interact with Number Language, indicating that the magnitude of a child's cross‐language counting difference did not modulate the size of their subitizing advantage in NL1 (see Supporting Information S1). Furthermore, an exploratory analysis found that these effects persisted when Highest Count was added to models (*p* < 0.05), suggesting that the effect of language was not due to different amounts of counting knowledge.

**FIGURE 2:**
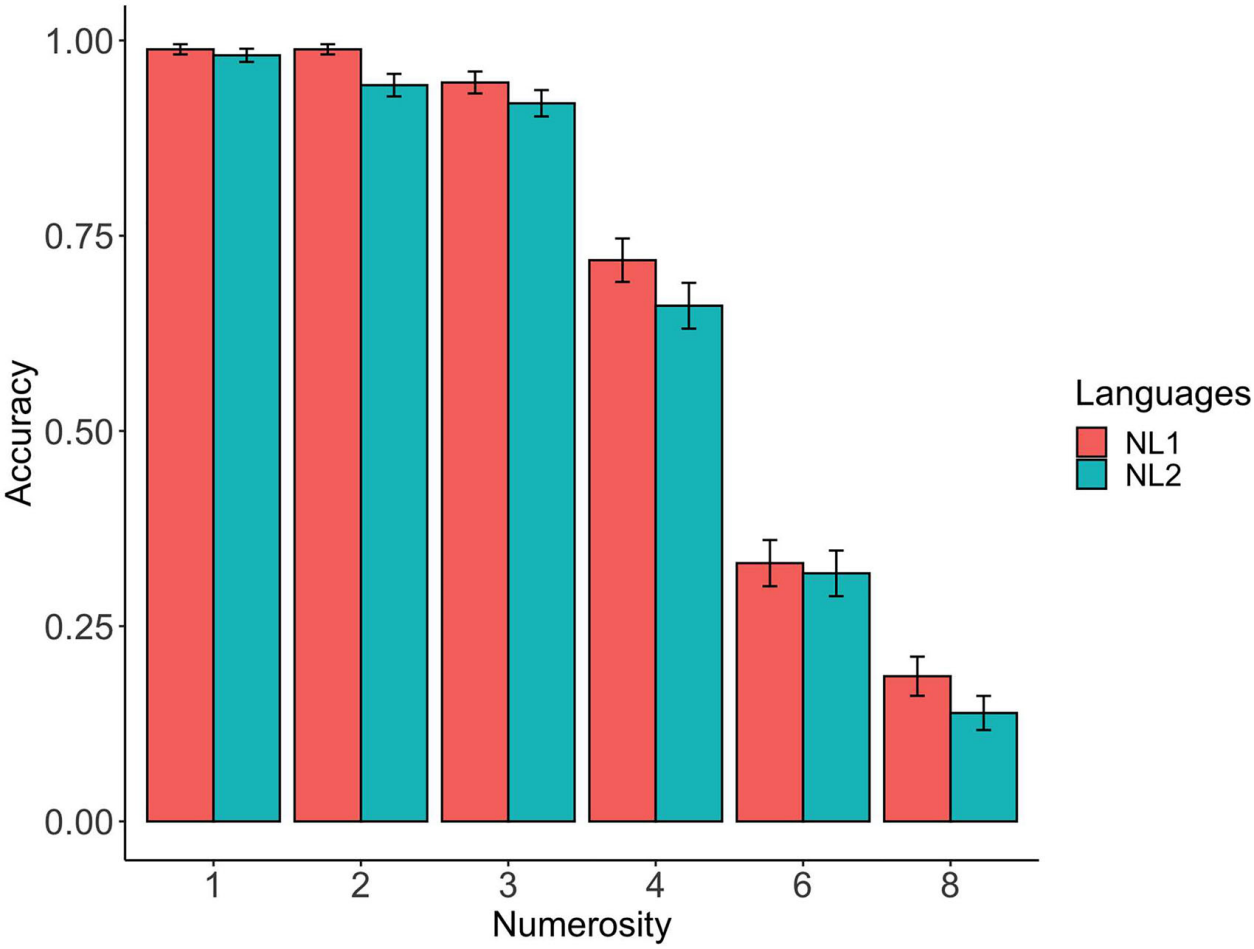
Accuracy at each numerosity in NL1 and NL2. *Note*: The *y*‐axis represents children's subitizing accuracy out of 4 trials for each numerosity. Numerosity is displayed on the *x*‐axis. NL1 and NL2, respectively, represent children's more and less dominant Number Language. Bars represent standard errors.

Next, following our pre‐registered plan, we constructed a third model in which we added the interaction between Number Language and Numerosity. This model found that Age, Numerosity, and Number Language remained significant (all *p*s < 0.05), though the interaction was not (*p* = 0.07). An ANOVA found that the addition of the Number Language × Numerosity interaction did not significantly improve model fit relative to our second model (*p* = 0.06). This suggests that the differences in subitizing accuracy between languages were somewhat uniform across numerosities, and that if differences existed, they were relatively small. Consistent with this, examining each set size individually (with Numerosity coded categorically) confirmed that NL1 accuracy exceeded NL2 at every set size in the subitizing range, though after Bonferroni correction only set size 2 exhibited a significant difference (see Supporting Information S4). A follow‐up analysis further confirmed that the Number Language effect did not depend on including set size 4 in the subitizing range (Supporting Information S4).

### 3.4 Exploratory Analyses

We next conducted a series of exploratory analyses to address: (1) whether the linguistically mediated differences in subitizing observed in bilingual children extended to larger numbers (i.e., 6 and 8) and (2) how the subitizing skills of monolingual children compared to those of bilingual children across their two languages.

#### 3.4.1 Estimation of Larger Numbers

In our first set of exploratory analyses, we asked whether bilinguals’ estimates for numbers outside the subitizing range differed across their two languages. To do this, we analyzed only responses provided for arrays of 6 and 8 dots, and used the same model comparison approach described in our main analyses section. In a model that predicted Accuracy from Age and Numerosity (and Participant as a random factor), both factors were significant (Age: *Estimate* = 0.59; SE = 0.18; *Z* = 3.27; *p* < 0.01; and Numerosity: *Estimate* = −0.58; SE = 0.09; *Z* = −6.65; *p* < 0.001). In a second model, we added the main effect of Number Language, but this factor was not significant, and the model did not improve fit to the data (both *p*s > 0.05). Similarly, the interaction between Numerosity and Number Language (model 3) did not add explanatory power to the first model (*p* > 0.05). Post hoc analyses found that this effect survived after the exclusion of children whose HC was below 8 (see Supporting Information S2). This suggests that Accuracy improved with Age and decreased with Numerosity similarly across languages. However, possibly explaining why no difference was found, as shown in Figure 2, the average accuracy for sets of 6 and 8 was overall very low (sets of 6:33% in NL1 and 32% in NL2; for sets of 8:19% in NL1 and 14% in NL2). This contrasts with data for small numbers, which was 91% overall in children's NL1 and 88% in their NL2, an issue we return to in Section 4. These null results held when the analysis was restricted to children who could count to at least 8 in both languages (Supporting Information S2), ruling out limited counting vocabulary as an explanation. To characterize performance beyond mere accuracy, we examined the full distribution of children's raw estimates for each numerosity and language (Figure 3). In the subitizing range (1–4), responses were tightly concentrated around the target. For the larger numerosities (6 and 8), estimates were approximately unbiased rather than systematically over‐ or under‐estimated (mean signed error between −0.41 and +0.44) and were broadly similar across languages. Finally, variability increased from the small number range (1–3) to the large number range (6 and 8). Together, these findings indicate that performance on sets of 6 and 8 reflected imprecise but unbiased estimation that did not differ across languages, converging with the accuracy results and supporting the floor‐effect interpretation (see Supporting Information S3 for the full analysis, including Table S3).

**FIGURE 3:**
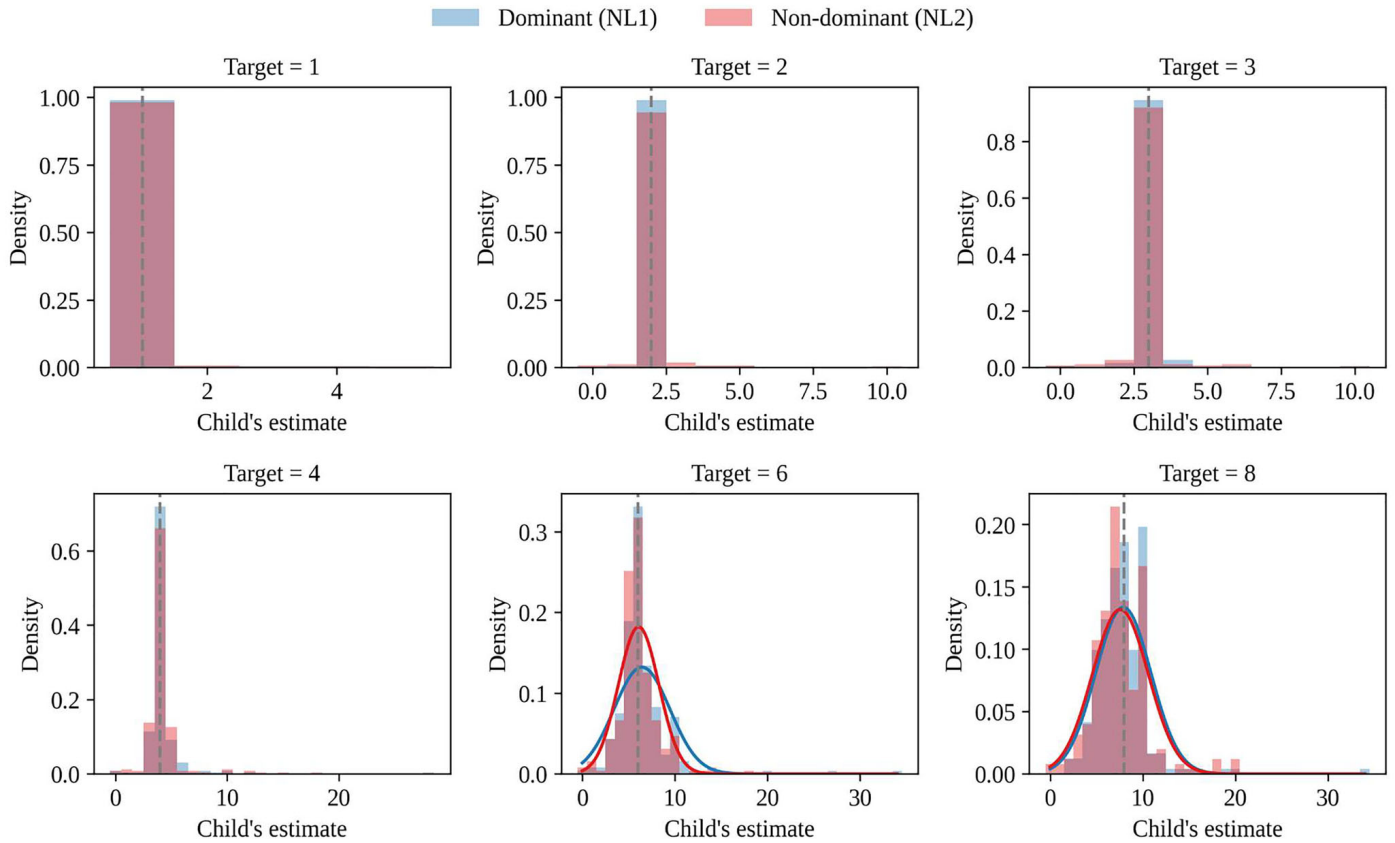
Distribution of children's estimates for each numerosity, separated by language (dominant vs. nondominant). *Note*. Dashed lines indicate target values; a fitted Gaussian is overlaid for large number targets (6 and 8), where Gaussian distributions are expected.

#### 3.4.2 Comparing Monolinguals and Bilinguals in Their NL1 and NL2

In our final exploratory analysis, we compared bilinguals’ subitizing skills to those of monolingual CP‐knowers. We did this for two reasons: first, to assess whether bilingual children show an overall delay in subitizing compared to monolingual children, and second, to assess how the mappings in bilinguals’ NL1 and NL2 compared to those of monolingual children who had the same knower level.^1^

We first excluded from the monolingual dataset all responses that were not in the format of a unique verbal estimate (*n =* 42/1008) or were 10 times larger than the mean estimate for each numerosity (*n =* 1/1008). The remaining 965 responses were compared to those of bilingual children in their NL1 and NL2. As in the main analyses, we restricted the dataset to responses for sets within the subitizing range of 1–4 dots.

We compared monolinguals' and bilinguals’ responses in NL1 and NL2 in two different sets of model comparisons.^2^ Table 1 shows the average response and Accuracy of monolingual children. To compare monolinguals' and bilinguals’ responses in their NL1, we first predicted Accuracy from Age and Numerosity (with Participant coded as a random factor) and found that both factors were significant (Age: *Estimate* = 1.37; SE = 0.29, *Z* = 4.78; *p* < 0.001; Numerosity: *Estimate* = −2.22; SE = 0.20, *Z* = −11.37; *p* < 0.001). In our second model, we added the main effect of bilingual status (either bilingual or monolingual) and found no significant effect of this factor (*p* = 0.36). This suggests that in our sample, the subitizing abilities of monolinguals and bilingual children in their NL1 did not differ. Next, to compare monolinguals and bilinguals’ NL2 responses, we predicted Accuracy from Age (*Estimate* = 1.45; SE = 0.31, *Z* = 4.68; *p* < 0.001) and Numerosity (*Estimate* = −1.85; SE = 0.15, *Z* = −12.32; *p* < 0.001), and in a second model, we added the effect of bilingual status. In this second model, all factors were significant (Age: *Estimate* = 1.63; SE = 0.32, *Z* = 5.11; *p* < 0.001; Numerosity: *Estimate* = −1.85; SE = 0.15, *Z* = −12.32; *p* < 0.001; Bilingual Status: *Estimate* = 1.05; SE = 0.47, *Z* = 2.22; *p* = 0.026). Since the main effect was significant, we added in a third model the interaction between Bilingual status and Numerosity, and this factor was also significant (*Estimate* = −0.78; SE = 0.34, *Z* = −2.28; *p* = 0.023) as were all others (Age: *Estimate* = 1.65; SE = 0.32, *Z* = 5.17, *p* < 0.001; Numerosity: *Estimate* = −1.63; *SE* = 0.17, *Z* = −9.82, *p* < 0.001; Bilingual status: *Estimate* = 3.84; *SE* = 1.34, *Z* = 2.88, *p* < 0.01). Taken together, these findings suggest that bilinguals who have comparable NL1 subitizing abilities to monolingual children nevertheless differ from monolinguals when considering their NL2. Finally, because the bilingual NL1 category collapses across English‐dominant and Other‐dominant (Spanish‐ or German‐dominant) children, we tested whether dominant‐language group affected subitizing accuracy by adding it to the main small‐number model. It did not (*Estimate* = 0.05, *p* = 0.90; likelihood‐ratio test *p* = 0.90), and the effect of Number Language was unchanged, indicating that our comparisons do not depend on which language was dominant.

**TABLE 1:** Average estimate and accuracy of response per Numerosity and Number Language in the fast cards task for monolingual children.

Numerosity	Responses		Accuracy	
	*M*	SD	*M*	SD
1	1.07	0.60	0.99	0.11
2	2.00	0.00	1.00	0.00
3	3.22	1.51	0.92	0.28
4	4.29	1.86	0.67	0.47

Abbreviations: *M*, mean; SD, standard deviation.

## 4 Discussion

In this study, we investigated language‐specific differences in children's representations of small number words by testing the subitizing abilities of bilingual CP‐knowers across their two languages. Specifically, we presented Spanish–English and German–English CP‐knowers with flashed arrays of small sets (1–4) and asked them to estimate these sets in each of their two languages. We found that bilingual children subitize differently across their two languages and specifically, that they are more accurate in the language in which they have the most experience counting. Also, we found that the subitizing abilities of bilinguals were comparable to those of monolinguals when considering their dominant number language, but that a difference did exist between groups when comparing bilingual children's nondominant number language to data from monolingual children. Finally, we found that bilinguals’ estimates for larger numbers did not differ across their languages, probably because accuracy for these numbers was overall very low. Taken together, these findings provide evidence that despite meeting the criteria to be classified as CP‐knowers in both languages, bilingual children still have different mappings between number words and small cardinalities across their two languages. This suggests that language‐specific experience plays an important role in the development of mappings between number words and cardinalities across languages, and that these differences persist even after children have progressed to later stages of development.

One hypothesis motivating the present study is that greater experience with counting in one language should strengthen the associative mappings between number words and their corresponding cardinalities in that language, resulting in more accurate subitizing responses. Given this idea, children should have stronger associations between number words and cardinalities in their dominant number language, in which they can count higher. However, alternative mechanisms are also possible. For example, another possibility is that bilingual children translate number words into a single dominant language before responding. However, contrary to our findings, this model would predict no difference in estimation accuracy across a child's two languages. Related to this alternative hypothesis, because we did not test response latency, future studies should explore whether children are faster to make estimates in their dominant number language, or if only accuracy differs across languages. Finally, language interference might occasionally lead children to retrieve a number word from the incorrect language, but it would not predict the asymmetric pattern we observed, namely, consistently greater accuracy in children's dominant number language.^3^

The results of this study allow us to integrate previous findings regarding number word learning and numerical estimation in bilingual children. In particular, our results suggest that the disparity in knowledge of number word meanings found in bilingual subset‐knowers (Alhaider et al. 2024; Sarnecka et al. 2018; Wagner et al. 2015) persists even after children learn to accurately count large sets and become CP‐knowers. This is compatible with previous findings that older bilingual children show differences in their estimation skills across languages (Marchand et al. 2020) and suggests that these differences begin to emerge from the early moments of number word learning. It is debated in the literature whether the processes of subitizing and the estimation of large numbers rely on the same underlying mechanisms; some argue that subitizing taps into a parallel individuation system—a system that keeps track of ∼ 4 individual items in parallel in working memory—while the second relies on the approximate number system (or Analog Magnitude System)—a system that allows us to apprehend approximately the magnitude of large sets (Carey 2004; Carey and Barner 2019; Feigenson et al. 2004; Hyde 2011). While our results can't directly address this issue, they provide evidence that language‐specific experience impacts both small and large numbers and that language‐related disparities emerge at an early age in bilingual children.

Our findings have implications for our understanding of number word learning in bilinguals, but are also relevant to theoretical debates regarding monolingual learners. Bilinguals’ successful application of the counting procedure in both languages, despite having different mappings across languages, raises questions about the causal relationship between learning the meanings of small number words and becoming a CP‐knower. According to bootstrapping accounts (e.g., Carey 2004, 2009), learning the meanings of small number words is an essential prerequisite to becoming a CP‐knower. In particular, on this account children notice that each time they add one object to a set, they should count up one word in the count list—a relation which, when generalized to all possible numbers, should allow them to accurately count and build large sets indefinitely. An alternative, however, is that learning to count is relatively independent of mapping small number words to their meanings, and that these are two distinct, though parallel processes (Barner, 2017). On this account, CP‐knowers initially perform the Give‐N task using rote procedures, and only gradually learn, through experience applying this procedure, how counting is related to iterative processes of addition. Although our data are hardly definitive, they are compatible with the idea that children can become CP‐knowers given relatively different degrees of knowledge of smaller number words, as argued by the “parallel process” model of number word learning. Our study also suggests that there may be individual differences in the strength of small number word knowledge between monolingual children who classify as CP‐knowers, and that possibly, some children may skip some subset stages altogether. Future studies should further explore this possibility.

These findings are also relevant to broader discussions of how estimation abilities develop. The overwhelming majority of previous studies have focused on large number estimation, and have found that estimation abilities are related to educational outcomes (Libertus et al. 2016; Koponen et al. 2016; Mazzocco et al. 2011; Schneider et al. 2018). By contrast, relatively little is known about how *subitizing* in the small number range relates to educational outcomes. Our findings reveal language‐related individual differences in subitizing, but leave open whether these early emerging differences are related to later learning outcomes. Future studies should explore this question and how early individual differences in bilingual learners are related to later math learning.

Beyond the primary findings reported in confirmatory analyses, we found two additional results in exploratory analyses: (1) cross‐linguistic differences found for small numerosities did not extend to larger sets (i.e., 6 and 8), and (2) bilingual children's subitizing skills in their NL1 were similar to the subitizing skills of monolinguals, unlike bilinguals’ subitizing skills in their NL2. With respect to the estimation of larger sets, the lack of a significant difference in accuracy is perhaps surprising given that older bilinguals show differences in estimation across languages (Marchand et al. 2020). However, one possible explanation for this discrepancy is that, because children in the current study were much younger than those in Marchand et al. (2020), they had not yet begun to form strong associative mappings for larger numbers in either language, and therefore failed to show linguistically mediated differences due to floor effects. As evidence for this, children's accuracy for sets of 6 and 8 was low, which contrasts with Marchand et al. (2020), in which the performance for these types of sets was much more accurate than for larger sets such as 60, 80, 98. With respect to the comparison of monolingual and bilingual children, our results suggest that bilinguals are neither delayed nor advanced relative to monolingual children. When tested in their dominant number language, bilinguals resembled monolingual English‐speaking children, whereas they performed slightly worse when tested in their nondominant language. This result corroborates findings from other studies with bilingual children that did not find differences between monolingual English speakers and Spanish–English children tested in English (i.e., bilinguals who attended preschool programs in English; Sarnecka et al. 2023).

These findings also underscore the importance of collecting L1 and L2 data from bilingual participants. Our bilingual participants' performance in their NL1 did not significantly differ from monolingual participants' performance. In contrast, studies of early numeracy in preschool‐aged bilingual children often test participants in a single language—typically their language of instruction—rather than in both their L1 and L2, despite the possibility that a young child's number‐word knowledge may be more closely tied to language spoken at home than to instructional language. This methodological choice may help explain some reports of weaker performance in bilingual children relative to monolinguals (e.g., Turan and De Smedt 2024). Rather than reflecting a comparative deficit in numerical cognition among bilingual children generally, such findings may instead reflect deficits that exist specifically in children's nondominant number language.

The current study also points to several directions for future work. First, while we found no important differences between our Spanish–English and German–English bilinguals, despite the greater relatedness of the latter two languages, it's possible that children learning very closely‐related languages (e.g., Spanish and Italian) might show no difference in subitizing abilities, or that much greater differences exist when languages differ more in form. Second, although children in our study were drawn from groups that likely differed in socio‐economic status, we did not measure these differences, leaving open whether effects of language dominance are greater in lower SES children. Relevant to this, past work has found that SES and immersion context are associated with which language becomes dominant for counting (e.g., higher‐SES L1 English‐speaking children in French immersion are more likely to be English‐dominant when tested on number knowledge, while lower‐SES L1 Spanish‐speaking children in English immersion are English‐dominant on numerical tasks. Crucially, in those past studies, and in our own, the central test consisted of a within‐child measure of numerical knowledge, such that SES could not explain effects of language within participants. Future work should nevertheless enrich this picture by incorporating a direct measure of SES, as well as a more detailed characterization of children's non‐numerical linguistic knowledge and their educational experiences. A further extension would be to include a nonverbal control measure of visuospatial ability, since our subitizing task likely has a visuospatial component that we were not able to isolate.

In sum, the current study shows that subitizing skills differ across bilingual children's two languages, such that they have stronger mappings in the language in which they can count the highest, despite being able to count beyond the small number range in both languages. These results have potentially important implications for mathematics education. In particular, researchers should not assume that bilinguals’ numerical skills are identical across their two languages. By the same token, apparent numerical deficits in bilingual children may in some cases reflect the language in which they were tested rather than their underlying competence, since testing outside a child's dominant number language can understate what they know. Children may reach a proficient level in math in their language of instruction, but may nevertheless be unable to apply this knowledge in their heritage language without additional language‐specific training. Future studies should investigate optimal methods for training these mappings in bilinguals, and whether it is better to explicitly teach children to translate across languages or instead offer separate language‐specific training of the mappings between small sets and their cardinalities.

## Author Contributions

**Kelly Kendro**: writing – original draft, writing – review and editing, investigation. **David Barner**: conceptualization, funding acquisition, investigation, methodology, formal analysis, software, project administration, writing – review and editing, writing – original draft, supervision, data curation, resources, validation, visualization. **Nina Schoener**: writing – original draft, writing – review and editing, investigation. **Jocelyn Sandoval‐Franquez**: writing – original draft, investigation. **Elisabeth Marchand**: conceptualization, investigation, funding acquisition, writing – original draft, methodology, validation, visualization, writing – review and editing, software, formal analysis, project administration, data curation, resources, supervision.

## Conflicts of Interest

The authors declare no conflicts of interest.

## Supporting information




**Supporting information**: desc70296‐sup‐0001‐SuppMat.docx

## Data Availability

All data and model outputs are available here: https://osf.io/gwz28/overview?view_only=0a7ad5625a544c3bbc30d1647cf862bb.

## Endnotes

1 Here, we deviate from the preregistered analyses to better fit our data, predicting Accuracy from Age, Numerosity (1–4), Number Language, and Highest Count in both NL1 and NL2. As reported, all factors were significant (all *p*s < 0.05). The values of Highest Count in NL1 and NL2 were also significantly correlated (*r*τ = 0.68, *p* < 0.001).

2 We originally planned to obtain a dataset of monolingual subset‐knowers to which we could compare the subitizing skills of bilingual children in their NL2. However, due to recruitment difficulty during the pandemic and the large sample size needed, we instead recruited monolingual CP‐knowers only.

3 An anonymous reviewer asked whether children ever responded using their non‐target language during testing. Such behaviors were rare in our study, and crucially, could not explain the pattern of results reported here, since any such responses would have been coded simply as incorrect.
