# Supplementary material for: The Development of Subitizing in Bilingual Children
Source: Dev Sci. 2026 Nov 3;30(1):e70296. doi: 10.1111/desc.70296 (PMC13731916; doi:10.1111/desc.70296)
Supplement: Supplementary file 1 [file DESC-30-e70296-s001.docx]

**Supplementary**

All supplementary analyses used the same mixed-effects logistic regression framework (*lme4*; Bates et al., 2015) and the same data-cleaning criteria described in the main text. Except where explicitly noted, these analyses were not preregistered and were conducted in response to reviewer feedback.

**S1: Continuous Dominance Score.** To verify that the effect of Number Language (with dominance treated as a categorical (NL1 vs. NL2) variable) did not depend on our binary classification of dominant versus non-dominant language, we computed two continuous dominance scores for each child: 1) the difference (NL1 - NL2) and 2) the ratio (NL1 / NL2) of their Highest Counts and added them to the second model. Neither improved fit as a main effect, and neither interacted with Number Language. Thus the magnitude of children's cross-language counting difference did not modulate the size of their subitizing advantage in NL1, and the effect of Number Language remained significant throughout.

**S2: Minimum Counting abilities.** Of the 66 bilingual children in our study, 8 had a Highest Count below 8 in at least one language; in every case this was the child’s non-dominant language, with the exception of one child who counted below 8 in both languages. No child counted below 4 in either language, so no exclusions were required for the subitizing-range (1–4) analysis. As a stricter check, excluding the one child whose *dominant*-language count fell below 8 left the Number Language effect essentially unchanged (*Estimate* = -0.60, *p* = .001). For the estimation range, restricting to the 58 children who counted to ≥8 in *both* languages left the 6-and-8 comparison non-significant (*p* = .33), with low accuracy across languages.

**S3. Error and response-distribution analysis.** Table S3 reports, for each target numerosity and language, the mean estimate, standard deviation, mean signed error (bias), and coefficient of variation (CV) underlying the response-distribution pattern summarized in the main text (see Figure 3). Within the subitizing range, precision (CV) was consistently better in children's dominant (NL1) than non-dominant (NL2) language (most notably at set size 2 (CV = 0.11 vs. 0.31)) whereas for the larger numerosities, CV was comparable across languages and across targets 6 and 8 (CV ≈ 0.36–0.47), consistent with the floor-effect interpretation.

**Table S3.** Number of responses, mean estimate, standard deviation (SD), mean signed error (bias; positive = over-estimation), and coefficient of variation by numerosity and language.

| **Target** | **Language** | ***n*** | **Mean estimate** | ***SD*** | **Bias** | **CV** |
| --- | --- | --- | --- | --- | --- | --- |
| 1 | NL1 (dominant) | 264 | 1.02 | 0.23 | +0.02 | 0.22 |
| 1 | NL2 (non-dominant) | 262 | 1.04 | 0.34 | +0.04 | 0.33 |
| 2 | NL1 (dominant) | 264 | 2.02 | 0.23 | +0.02 | 0.11 |
| 2 | NL2 (non-dominant) | 262 | 2.06 | 0.63 | +0.06 | 0.31 |
| 3 | NL1 (dominant) | 260 | 3.04 | 0.55 | +0.04 | 0.18 |
| 3 | NL2 (non-dominant) | 261 | 3.02 | 0.69 | +0.02 | 0.23 |
| 4 | NL1 (dominant) | 263 | 4.22 | 1.85 | +0.22 | 0.44 |
| 4 | NL2 (non-dominant) | 262 | 4.22 | 1.75 | +0.22 | 0.41 |
| 6 | NL1 (dominant) | 254 | 6.44 | 3.02 | +0.44 | 0.47 |
| 6 | NL2 (non-dominant) | 255 | 6.08 | 2.19 | +0.08 | 0.36 |
| 8 | NL1 (dominant) | 242 | 7.91 | 2.99 | −0.09 | 0.38 |
| 8 | NL2 (non-dominant) | 252 | 7.59 | 3.03 | −0.41 | 0.40 |

**S4. Numerosity as a categorical factor.** At a reviewer's suggestion we re-fit the main model with Numerosity as a categorical factor. In the main analyses, Numerosity was modeled as a continuous predictor because we had no *a priori* hypotheses about individual set sizes. The categorical analyses reported here were included in our preregistered *post hoc* plan.

**S4a. Per-numerosity comparison across languages.** We re-fit the main small-number model with Numerosity as a categorical factor interacting with Number Language and compared NL1 and NL2 accuracy at each set size (1–4), applying a Bonferroni correction across the four comparisons (Table S4). The NL1 advantage was significant only at set size 2 (NL1 = 98.9%, NL2 = 94.3%; *p* = .027). Differences at set sizes 1, 3, and 4 were not significant, although accuracy was slightly higher in NL1 than NL2 at every set size. This pattern is consistent with the main analyses. At set size 1, accuracy was near ceiling in both languages (98.9% vs. 98.1%), leaving little room to detect a language difference. At set sizes 3 and 4, accuracy was lower and more variable. The significant effect at set size 2 therefore likely reflects the combination of near-ceiling performance in NL1 and a detectable decline in NL2, rather than a mechanism specific to that numerosity. Overall, these results converge with the main analyses, indicating a modest NL1 advantage across the 1 to 4 number range that is difficult to detect at individual set sizes, particularly after the conservative Bonferroni correction.

**S4b. Differences among set sizes.** To test whether accuracy differed across the individual set sizes (i.e., as distinct from the language comparisons above) we computed all pairwise comparisons among numerosities from the same model, averaged over language and Bonferroni-corrected across the six comparisons. Accuracy did not differ between set sizes 1 and 2, which were both near ceiling (*p* = 1.00), but declined significantly at set size 3 relative to both set size 1 (*p* < .001) and set size 2 (*p* = .029), and dropped most sharply at set size 4, which differed from every smaller set size (all *p*s < .001). Performance was thus not uniform across the subitizing range: the smallest sets (1 and 2) were statistically indistinguishable, whereas accuracy fell progressively at set sizes 3 and 4.

**S4c. Robustness to the definition of the subitizing range.** Although we observed a steep decline at set size 4 relative to smaller sets (S4b), accuracy at set size 4 (NL1 = 71.9%, NL2 = 66.0%) remained far higher than for larger sets (sets of 6: 33% and 32%; sets of 8: 19% and 14% in NL1 and NL2, respectively), indicating that set size 4 patterned more closely with the subitizing range rather than behaving as an estimation item. However, to confirm that our main conclusions about subitizing do not depend on the inclusion of set size 4, we re-ran the main analysis on set sizes 1–3 only. The effect of Number Language remained significant (*Estimate* = −0.91, *SE* = 0.30, *z* = −2.97, *p* = .003) and significantly improved model fit (*χ*²(1) = 9.38, *p* = .002). The NL1 subitizing advantage therefore does not hinge on including set size 4.

**Table S4.** Accuracy by numerosity and language (subitizing range), with the NL2 − NL1 contrast (log-odds) and Bonferroni-corrected *p*-value at each set size.

| **Numerosity** | **NL1 accuracy** | **NL2 accuracy** | **NL2 − NL1 (log-odds)** | **Corrected *p*** |
| --- | --- | --- | --- | --- |
| 1 | 98.9% | 98.1% | −0.57 | > .99 |
| 2 | 98.9% | 94.3% | −1.78 | .027 |
| 3 | 94.6% | 92.0% | −0.50 | .76 |
| 4 | 71.9% | 66.0% | −0.43 | .29 |
